# *MSTN* Mutant Promotes Myogenic Differentiation by Increasing Demethylase *TET1* Expression via the SMAD2/SMAD3 Pathway

**DOI:** 10.7150/ijbs.40551

**Published:** 2020-02-21

**Authors:** Li Gao, Miaomiao Yang, Zhuying Wei, Mingjuan Gu, Lei Yang, Chunling Bai, Yunxi Wu, Guangpeng Li

**Affiliations:** 1 State Key Laboratory of Reproductive Regulation & Breeding of Grassland Livestock, Inner Mongolia University, Hohhot, 010070, China; 2 School of Life Science, Inner Mongolia University, Hohhot, 010070, China

**Keywords:** *MSTN* mutant, myogenic differentiation, ten-eleven translocation methylcytosine dioxygenase 1 (*TET1*), SMAD2/SMAD3, DNA methylation

## Abstract

Myostatin (*MSTN*) is mostly expressed in skeletal muscle and plays crucial roles in the negative regulation of muscle mass development. The methylation and demethylation of myogenesis-specific genes are major regulatory factors in muscle satellite cell differentiation. The present study was designed to investigate the mechanism of myogenic differentiation regulated by *MSTN* mutation (MT) and the methylation/demethylation state of downstream genes. The results showed that, in the *MSTN*^-/+^ satellite cells, a higher myotube fusion index and a larger myotube length were observed compared to the wild type controls; the genes associated with myogenesis were all up-regulated compared to the WT controls. The methylation of the promoters and gene bodies of *PAX3*, *PAX7*, *MyoD*, and *MyoG* were all down-regulated, while the expression of the key demethylase *TET1* was significantly promoted. ChIP-qPCR was used to demonstrate that the SMAD2/SMAD3 complex combined with the promoter of *TET1* to inhibit the activity of *TET1* promoter, indicating that *MSTN* may regulate *TET1* via SMAD2/SMAD3. The overexpression of *TET1* in wild type cells promoted myogenic differentiation, increased the myotube index, and reduced the methylation of the associated genes. On the contrary, the knockdown of *TET1* in the *MSTN* mutant cells resulted in the opposite phenomena as in the overexpressed cells. In conclusion, the myostatin mutant showed an increased transcriptional activity of *TET1*, inducing higher levels of demethylation and improving the transcriptional activity levels of myogenic differentiation-associated genes. The binding of SMAD2/SMAD3 directly to the *TET1* promoter region indicated that the *MSTN* mutant demethylated the myogenesis-specific genes by up-regulating *TET1*, which is directly controlled by SMAD2/SMAD3.

## Introduction

Myostatin (*MSTN*), also known as growth/ differentiation factor-8, is a member of the transforming growth factor β (TGF-β) family; it is mostly expressed in skeletal muscle and plays a crucial role in the negative regulation of muscle mass development 1. Genetic studies have demonstrated that *MSTN* deficiency leads to muscle hypertrophy due to a combination of increased myofiber numbers and increased myofiber sizes in multiple species, including humans, sheep, dogs and cattle 1-5, without causing severe adverse consequences. Therefore, extensive efforts have been made to develop effective strategies for blocking the expression of *MSTN* in order to increase the muscle mass of animals. Luo *et al.* (2014) reported on the production of *MSTN* knockout cattle mediated by Zinc finger nuclease 6, Wang *et al.* (2017) produced *MSTN* knockout pigs by CRISPR/Cas9 7, Yu *et al.* (2016) 8 and Wang *et al.* (2018) 9 produced *MSTN* knockout goats by TALEN and CRISPR/Cas9, and Zhang *et al.* (2018) produced a fat-1 transgenic goat integrated at the *MSTN* locus. All of these *MSTN* knockout animals showed improved skeletal muscle development 10.

Epigenetic events regulate the quiescent and proliferation state of muscle satellite cells and their progeny. DNA methylation is a major repressive mechanism of muscle satellite cell differentiation 11, 12, whereas demethylation, along with *MyoD* and myogenin, are required for the initiation of the differentiation program 11. Previous studies have proven that the CpG islands of *MYF5* undergo hypomethylation during myogenic differentiation 13. The modulation of methylation via prolonged treatment with 5-azacytidine, an inhibitor of DNA methylation, has been associated with an increased myogenic commitment of fibroblasts, mature adipocyte-derived dedifferentiated fat cells, and cardiac cells 14-17. Furthermore, in C2C12 cells, treatment with 5-azacytidine resulted in an enhanced expression of muscle-specific genes (including myogenin) and increased myotube maturation, which suggested that the inhibition of methylation was a suitable tool to further boost myogenic differentiation in already committed precursors 18, 19.

Although both *MSTN* and DNA methylation are related to skeletal muscle development, the mechanism of *MSTN* and DNA methylation in muscle satellite cell differentiation remains unclear. In the present study, muscle satellite cells derived from *MSTN* mutant and wild type cattle were isolated and myogenic differentiation was induced. The myotube index including fusion rate and myotube length, expression and methylation levels of genes associated with myogenesis, and the expression of demethylase TETs were investigated. Our findings provide important insights into the roles of *MSTN* in epigenetic modification during myogenesis.

## Results

### Isolation and identification of the muscle satellite cells from *MSTN* mutant and wild type cattle

**Figure 1:**
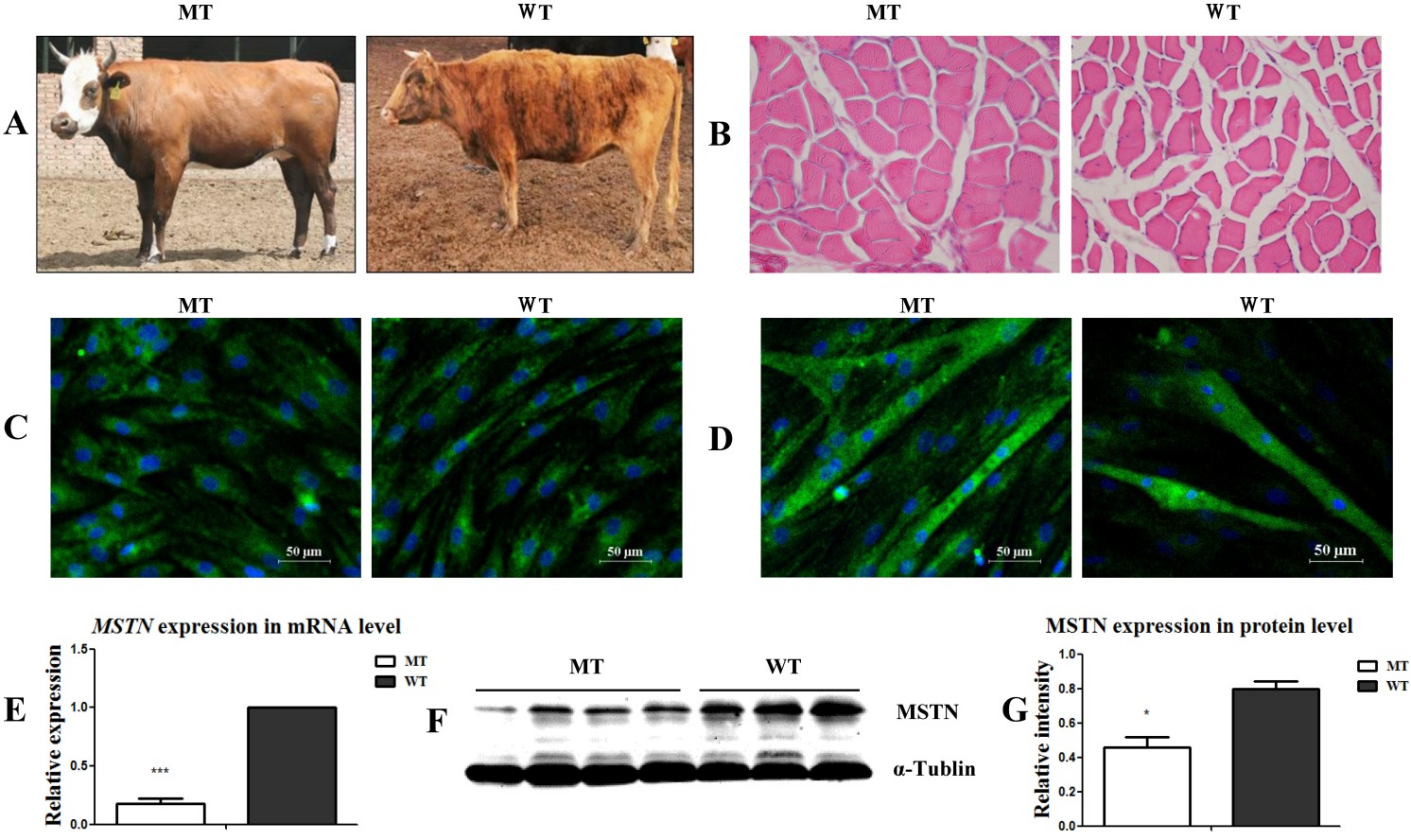
** Identification of the muscle satellite cells from *MSTN* mutant (MT) and wild type (WT) cattle. (A)** Samples from MT and WT cattle showing different muscular morphologies. **(B)** Cross-sectional images of muscle tissues from MT and WT cattle (200×). **(C)** Identification of satellite cells with MyoD expression. **(D)** Identification of differentiated myotubes with the expression of MHC. **(E)** Expression of *MSTN* mRNA in the satellite cells. (F, G) Expression of MSTN protein in satellite cells. *P<0.05, **P<0.01, ***P<0.001; t-tests were used to calculate the p values.

As shown in Figure 1A, the *MSTN* mutant (MT) cattle had a visibly stronger muscle morphology than the wild type (WT) cattle. The muscle myofiber cross-sectional areas (CSA) of the longissimus dorsi muscles in the MT cattle were larger than those in the WT cattle (Figure 1B). After culturing the muscle tissues for 4 days, the presumptive satellite cells were obtained. Cell identification showed that the MSCs markers CD90 and CD105 were positively expressed, while the hematopoietic lineage marker CD34 was negatively expressed (Figure S1A) in both the MT- and WT-derived cells. Further identification of the cells by differentiation induction showed that both the MT and WT cells positively expressed the myogenic markers of MyoD (Figure 1C) and MHC (Figure 1D) during myogenic differentiation. The expression of MSTN at both the mRNA (Figure 1E) and protein (Figure 1F, 1G) levels significantly decreased in the MT-derived satellite cells compared to that in the WT cells. The presumptive satellite cells also had the capacity for adipogenic and osteogenic differentiation (Figure S1B, S1C). These results confirm that the isolated cells were *MSTN* mutant (MT) and wild type (WT) cattle muscle satellite cells.

### *MSTN* mutant promoted myogenic differentiation and increased the expression of myogenic genes

**Figure 2:**
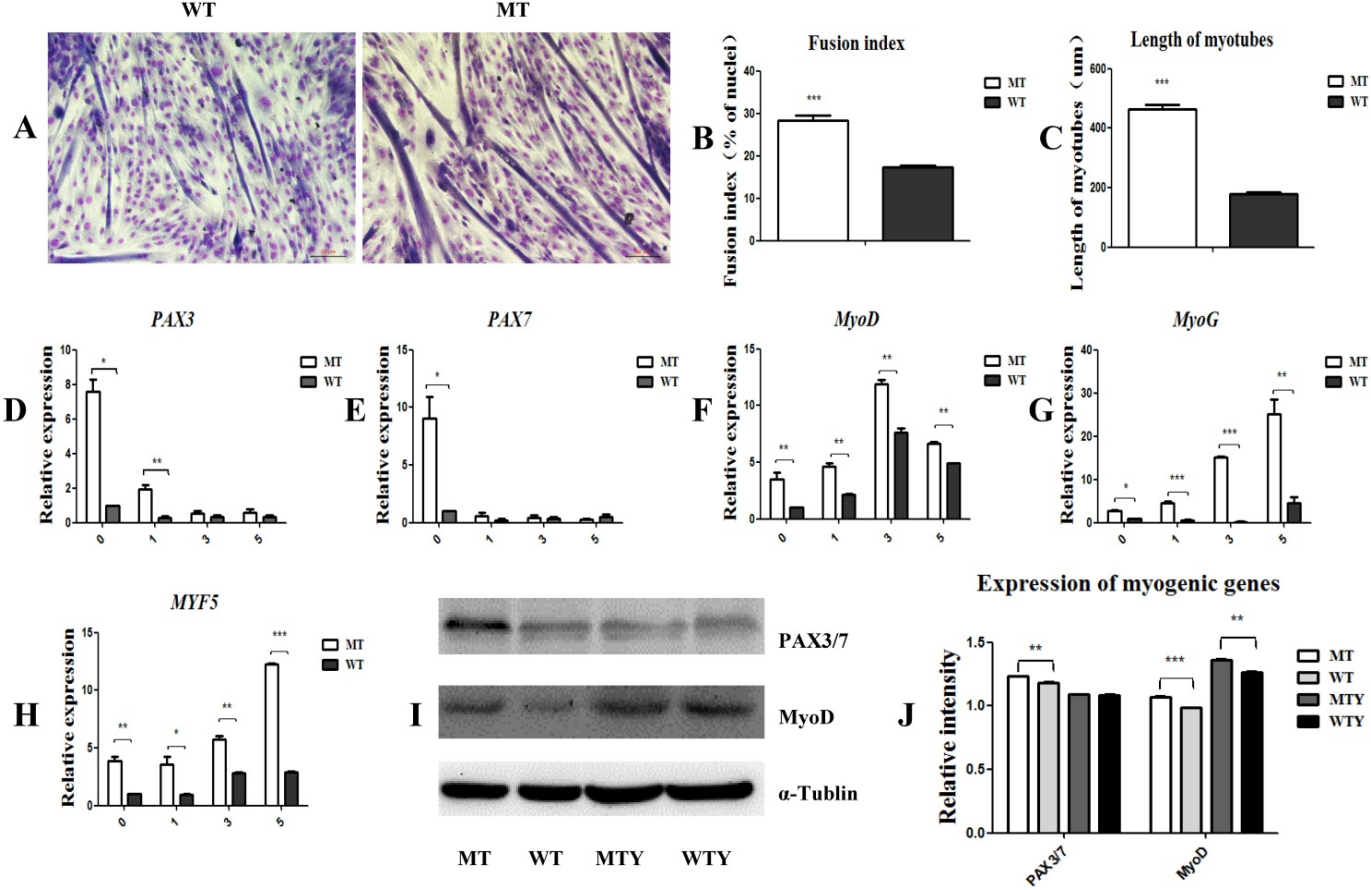
***MSTN* mutant promotes myogenic differentiation and increases the expression of myogenic differentiation-associated genes. (A)** Differentiated myotubes in both MT and WT cells. **(B)** Fusion index. **(C)** Length of myotubes. **(D-H)** mRNA levels of myogenic-associated genes *PAX3*, *PAX7*, *MyoD*, *MyoG*, and *MYF5* in the differentiated MT and WT muscle satellite cells. **(I, J)** Protein expression of PAX3/7 and MyoD genes in the differentiated MT and WT muscle satellite cells. WTY: Myotubes induced from WT cells; MTY: Myotubes induced from MT cells. *P<0.05, **P<0.01, ***P<0.001; t-tests were used to calculate the p-values.

Myogenic differentiation was induced in the satellite cells of both the MT and WT cattle. Three days later, the cells fused to form myotubes with a fusion index of 28.2% in the MT cells, which was significantly higher than that of the WT satellite cells (17.3%). The length of myotubes of the MT cells was higher than that of the WT cells (446.3892 ± 55.5 vs 117.4693 ± 24.1; P<0.05) (Figure 2A, 2B, and 2C). To further detect the expression of genes associated with myogenic differentiation, cells induced for 1, 3, and 5 days were collected, respectively, and the mRNA was extracted. *PAX3*, *PAX7*, *MyoD*, *MyoG*, and *MYF5* were up-regulated in the MT satellite cells compared to those in the WT controls (Figure 2D-2J). These results indicate that the *MSTN* mutant promoted the myotube fusion index, elongated the myotube length, and increased the expression of the myogenic differentiation-associated genes.

### *MSTN* mutant demethylated myogenesis-specific genes by up-regulation of demethylase *TET1*

**Figure 3:**
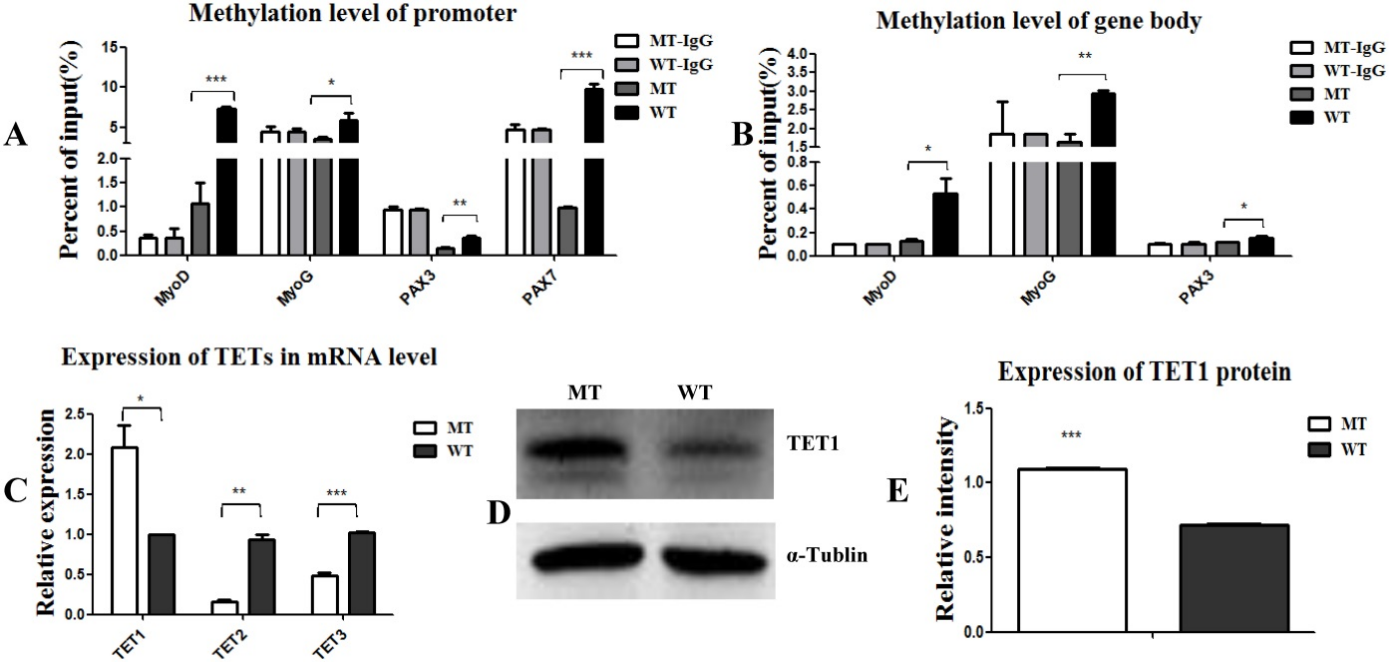
***MSTN* mutant demethylates myogenesis-specific genes by up-regulating demethylase *TET1*. (A, B)** Methylation levels of myogenesis-specific genes of *MyoD*, *MyoG*, *PAX3*, and *PAX7* in promoters (A) and gene bodies (B). **(C)** Expression of demethylase *TET1*, *TET2*, and *TET3* at the mRNA levels. **(D, E)** Expression of demethylase TET1 at the protein level. *P<0.05, **P<0.01, ***P<0.001; t-tests were used to calculate the p-values.

Epigenetic events, such as DNA methylation and demethylation of *MyoD*, *MYF5*, and *MyoG*, are known to play important roles in the regulation and initiation of differentiation 11, 13. Here, we aimed to examine the methylation state of *PAX3*, *PAX7*, *MyoD*, and *MyoG*. As shown in Figure 3, the methylation levels of the promoters and gene bodies of *PAX3*, *PAX7*, *MyoD*, and *MyoG* were all down-regulated (Figure 3A, 3B), suggesting that the *MSTN* mutant demethylated myogenesis-specific genes, thus promoting the expression of these genes. To confirm this demethylation, we analyzed the activity of the demethylases *TET1*, *TET2*, and *TET3* and found that *TET1* expression was promoted, while *TET2* and *TET3* expression decreased (Figure 3C, 3D, and 3E). These results demonstrate that the *MSTN* mutant demethylated the myogenesis-specific genes by up-regulating demethylase *TET1*, thus promoting myogenic differentiation.

### *MSTN* regulated *TET1* expression via SMAD2/SMAD3

**Figure 4:**
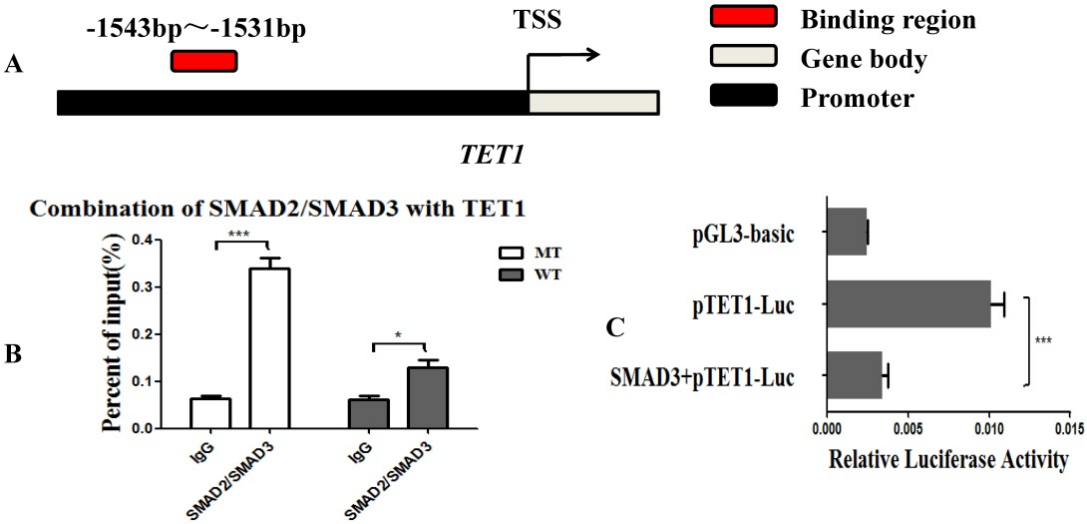
***MSTN* regulates *TET1* expression via SMAD2/SMAD3. (A)** The binding region of SMAD2/SMAD3 with the promoter of *TET1*. **(B)** The combination of *TET1* promoter with SMAD2/SMAD3 detected by ChIP-qPCR. The detected binding region was from ‒1613 to ‒1437 on the *TET1* promoter. **(C)** Luciferase reporter assays. HEK293T cells were transfected with luciferase reporter plasmids containing the promoter of *TET1* (1800 bp). *P<0.05, **P<0.01, ***P<0.001; t-tests were used to calculate the p-values.

Since the *MSTN* mutant improved the transcriptional activity of *TET1*, we hypothesized that the SMAD2 and SMAD3 transcription factors, which are found downstream of the *MSTN* type II receptors, may bind with the promoter of *TET1* and regulate the transcriptional activity of *TET1*. We used the JASPAR database (http://jaspar.binf.ku.dk/cgi-bin/jaspar_db.pl) for prediction analysis and found that SMAD2/SMAD3 bound to the *TET1* (NC_037355.1) promoter region from ‒1543 to ‒1531 (Figure 4A, Figure S2A). To confirm this, we performed a ChIP assessment using an anti-SMAD2/SMAD3 monoclonal antibody with an amplified *TET1* binding area. The detected regions spanned from ‒1613 to ‒1437 on the *TET1* promoter. The ChIP-qPCR results showed that SMAD2/SMAD3 directly binds to the promoter of *TET1* (Figure 4B, Figure S2B). To study the effect of SMAD2/SMAD3 on the transcriptional activity of *TET1*, we detected the transcriptional activity of *TET1* using a Luciferase reporter assay after the overexpression of *SMAD3*. *SMAD3* was found to inhibit the transcriptional activity of the *TET1* promoter (Figure 4C), and the overexpression of *SMAD3* was found to inhibit the expression of *TET1* at both the mRNA and protein levels (Figure S3).

### Overexpression of *TET1* promoted myogenic differentiation

**Figure 5:**
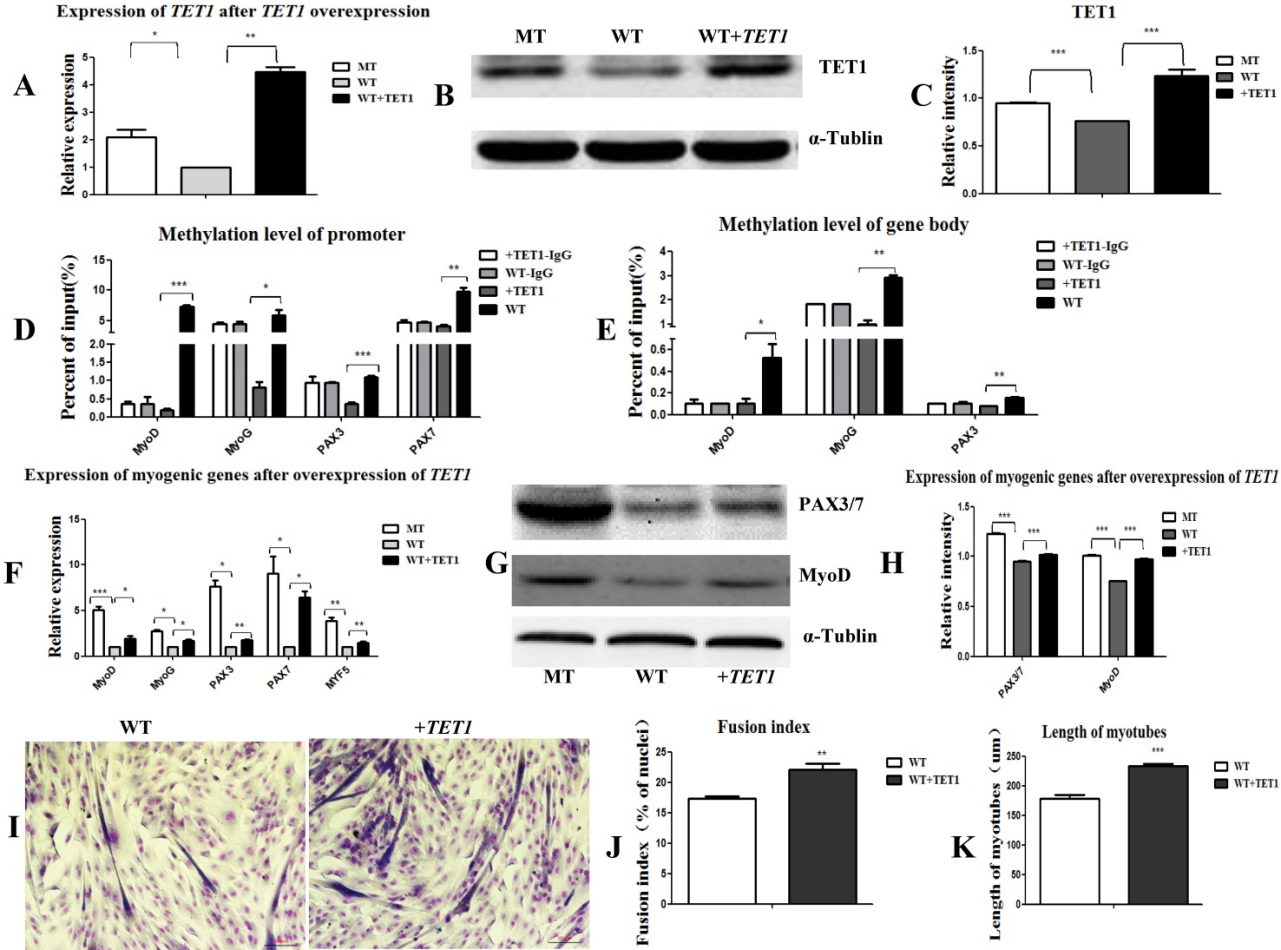
** Overexpression of *TET1* promotes myogenic differentiation. (A-C)** The expression of TET1 at the mRNA and protein level after the overexpression of *TET1* in the wild type muscle satellite cells. **(D, E)** Methylation levels of myogenesis-specific genes in the promoter and gene body. **(F)** Expression of myogenic genes at the mRNA level. **(G, H)** Expression of myogenic genes at the protein level. **(I)** The differentiated myotubes. **(J)** Myotube fusion index after the overexpression of *TET1*. **(K)** Length of myotubes. *P<0.05, **P<0.01, ***P<0.001; t-tests were used to calculate the p-values.

In the following experiment, we transfected the wild type satellite cells with the *TET1* overexpression vector (Figure S4B) to further prove the regulatory role of *TET1* in myogenic differentiation. As shown in Figure 5A, 5B, and 5C, the mRNA and protein expression levels of *TET1* in the transgenic cells increased significantly. The methylation levels in the promoters (Figure 5D) and gene bodies of *MyoD*, *MyoG*, *PAX3*, and *PAX7* (Figure 5E) decreased significantly. The mRNA and protein levels of the above genes were significantly promoted (Figure 5F, 5G, and 5H). After the induction of myogenic differentiation, the myotube fusion index (Figure 5I, 5J) was higher and the length of myotubes (Figure 5K) was larger in the *TET1* overexpressed cells than in the controls.

### Knockdown of *TET1* inhibited myogenic differentiation

**Figure 6:**
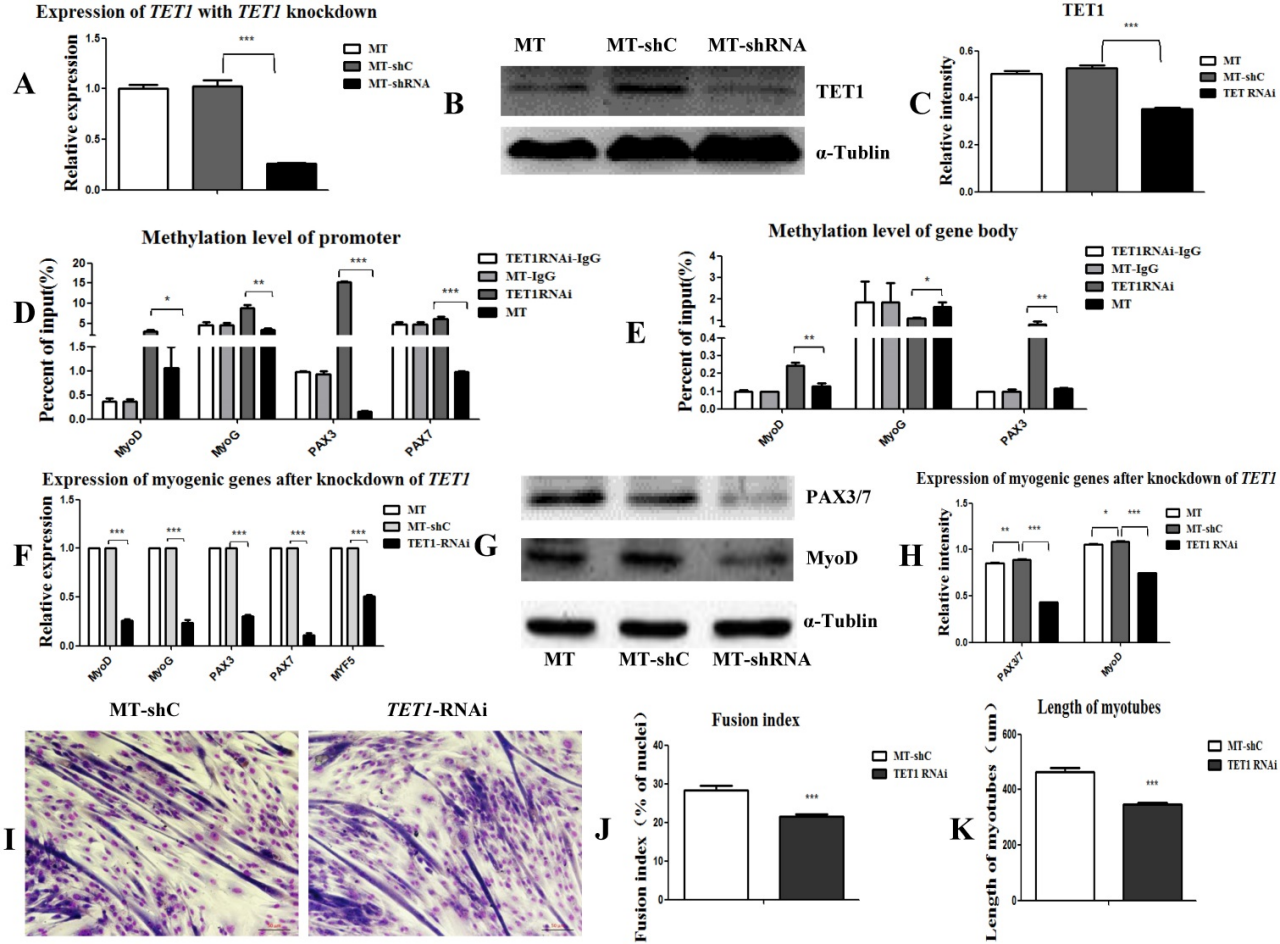
** Knockdown of *TET1* inhibits myogenic differentiation. (A-C)** The expression of TET1 at the mRNA and protein level after knockdown of *TET1* in *MSTN* mutant muscle satellite cells. **(D, E)** Methylation level of myogenesis-specific genes in promoter and gene body. **(F)** Expression of myogenic genes at the mRNA level. **(G, H)** Expression of myogenic genes at the protein level. **(I)** The differentiated myotubes. **(J)** Myotube fusion index after *TET1* knockdown. **(K)** Length of myotubes. *P<0.05, **P<0.01, ***P<0.001; t-tests were used to calculate the p-values.

The overexpression of *TET1* increased the myogenic differentiation and the expression of the associated genes. For the knockdown of *TET1*, we used satellite cells derived from the *MSTN* mutant muscle as target cells for transfection with *TET1* shRNA. The results showed that the expression of *TET1* at both mRNA and protein level decreased significantly (Figure 6A, 6B, and 6C). The methylation in the promotors (Figure 6D) and gene bodies (Figure 6E) of *MyoD*, *MyoG*, *PAX3*, and *PAX7* was significantly promoted after the knockdown of *TET1* in the mutation cells. The expression of these genes at both the mRNA and protein level was significantly decreased (Figure 6F, 6G, and 6H). The myotube fusion index was also significantly decreased (Figure 6I, 6J) and the length of the myotubes was shorter after *TET1* knockdown (Figure 6K).

## Discussion

*MSTN* is known to negatively regulate muscle development 1. As a result, *MSTN* mutation leads to muscle hypertrophy growth, as reported previously in beef cattle 2, humans 3, goats 4, and dogs 5, resulting from a combination of an increase in the number and size of myofibers 1-5. Since muscle mass is an important indicator in animal production, extensive efforts have been made to develop effective strategies that block the expression of *MSTN* for the production of animals with increased muscle masses 20. Several studies have demonstrated that the overexpression of *MSTN* inhibits the myogenic process by downregulating the mRNA levels of muscle regulatory factors *MyoD* and *MyoG* 21, 22. In contrast, the shRNA knockdown of endogenous *MSTN* results in the induction of myogenic differentiation 22. Previous studies have also shown that the overexpression of *MSTN* inhibits *SMAD3* and *MyoD* activity, resulting in the failure of myoblasts differentiate into myotubes 23. In the present study, we isolated muscle satellite cells from *MSTN* mutant and wild type cattle. The *MSTN^ -/+^* satellite cells were found to have a higher myotube fusion index and larger myotube length than the control cells. The expression of genes associated with myogenic differentiation, such as *MyoD*, *MyoG*, *PAX3*, and *PAX7*, were also significantly higher in the *MSTN^ -/+^* satellite cells. These results indicate that the *MSTN* mutation increases the rate of muscle development by increasing the levels of myogenesis-specific gene expression.

DNA methylation is associated with gene silencing 24. The levels of methylation are catalyzed by specific methyltransferases (DNMT) and demethylases (TET) 25, 26. Various studies have demonstrated that DNA methylation is a major repressive mechanism of muscle satellite cell differentiation 11, 13, whereas demethylation, as well as *MyoD* and *MyoG*, are required for the initiation of the differentiation program 11. In C2C12 cells, treatment with 5-azacytidine resulted in an enhanced expression of muscle-specific genes (including myogenin) and increased myotube maturation 18, 19, which suggests that DNA methylation plays an important role in the regulation of the differentiation of muscle satellite cells. In the present study, we found that the* MSTN* mutant promoted the expression of the demethylase *TET1*, but reduced the expression of *TET2* and *TET3*. The methylation levels of myogenesis-specific genes were all decreased, especially in the promoter region. These results indicate that the myogenesis-specific genes *MyoD*, *MyoG*, *PAX3*, and *PAX7* may be regulated by *TET1*. Additionally, previous studies have demonstrated that TETs play important roles in the regulation of muscle development 27-29 via the demethylation of myogenesis-specific genes. *TET1* and *TET2* were found to meditate the conversion of methylated cytosine into 5hmC, which is the first step in the DNA demethylation active pathway 30-32. However, the overexpression of *TET3* was found to not cause an obvious decrease in 5mC staining 33. In this study, the expression of *TET1* was up-regulated and the expression of *TET2* and *TET3* were down-regulated in the *MSTN* mutant cells. This may be due to the fact that the gene body region had low levels of methylation, such that the demethylation of myogenic genes mainly occurred in the promoter region. *TET1* meditates the conversion of methylated cytosine into 5hmC, particularly in the promoter region 34, such that the demethylation of the *MSTN* mutant was mainly carried out by *TET1*. By contrast, *TET2* and *TET3* did not play an important role in this process. However, the reason for the down-regulation of *TET2* and *TET3* will require further study.

To further evaluate the role of *TET1* during myogenic differentiation, *TET1* was overexpressed in the muscle satellite cells of the wild type cattle. The results showed that the methylation levels of myogenesis-specific genes were significantly decreased, while the expression of these genes was visible increased and the myogenic differentiation was promoted. However, upon the knockdown of *TET1* in the *MSTN* mutant muscle satellite cells, the methylation of the myogenic genes increased, while the expression levels of these genes were markedly reduced and the myogenic differentiation inhibited. These results further demonstrate that the *MSTN* mutant promoted myogenic differentiation via the demethylation of myogenesis-specific genes, particularly in the promoter region, via the up-regulation of *TET1*.

Since SMAD2 and SMAD3 are known to be downstream of the *MSTN* type II receptors, the combination of *MSTN* with its receptors results in the phosphorylation and activation of SMAD2 and SMAD3 23, 35, 36. As such, we hypothesized that the SMAD2/SMAD3 transcription factors are likely to interact directly with *TET1*. We predicted the binding of SMAD2/SMAD3 to the *TET1* promoter region using the JASPAR database and performed ChIP assays with an anti-SMAD2/SMAD3 monoclonal antibody. Using ChIP-qPCR, we found that SMAD2/SMAD3 was able to bind with the promoter of *TET1*. Moreover, a Luciferase reporter assay indicated that the overexpression of *SMAD3* inhibited the transcriptional activity of *TET1*. In the present study, the *MSTN* mutant was found to result in decreased SMAD2/SMAD3 activity 23, 35, which reduced the inhibition of SMAD2/SMAD3 by the transcriptional activity of *TET1*, leading to increased levels of *TET1* activity. These increased levels of *TET1* activity resulted in the demethylation of the myogenesis-specific genes, thereby promoting myogenic differentiation.

**Figure 7:**
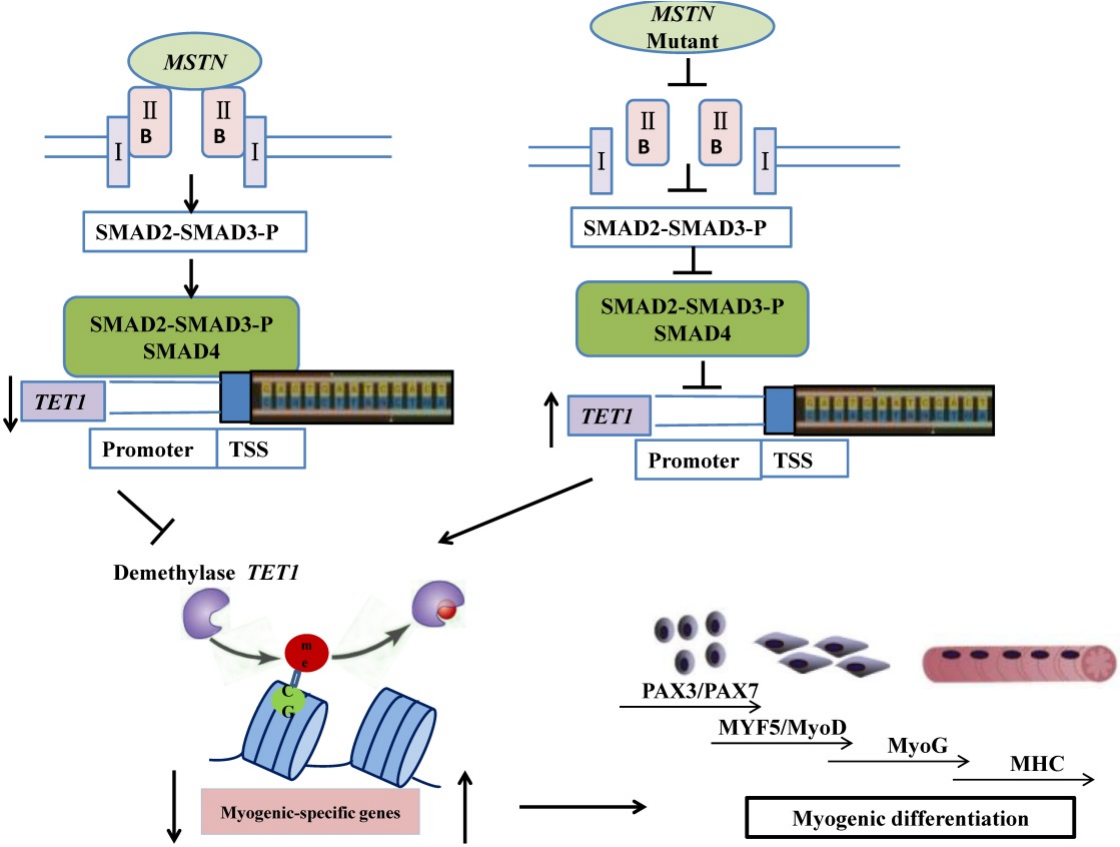
** Schematic of mechanisms for the inhibition of SMAD2/SMAD3 and the activation of *TET1* signaling by *MSTN* mutation.** In the wild type model (left), *MSTN* combined with ActRIIB, followed by the combination of the complex with type I receptor, the activation of the GS region (Ser/Thr) of the receptor, and the transmission of the *MSTN* signal. The activated *MSTN* signal then combined and phosphorylated SMAD2/SMAD3, whereby pSMAD2/SMAD3 was activated and combined with SMAD4. This complex entered the nucleus to combine with the promoter of demethylase *TET1*, and inhibited the activity of *TET1* promoter. In the *MSTN* mutant model (the right), the *MSTN* mutant decreased the binding between pSMAD2/SMAD3 and the promoter of *TET1* and enhanced *TET1* expression, which increased the demethylation of myogenesis-specific genes, including *PAX3*,* PAX7*, *MyoD*, and *MyoG*.

In conclusion, the mutation of myostatin resulted in increased levels of *TET1* activity, which induced higher levels of demethylation and myogenic-associated gene activity. SMAD2/SMAD3 bound directly to the *TET1* promoter region, which indicated that the *MSTN* mutant demethylated the myogenesis-specific genes by up-regulating *TET1*, which is directly controlled by SMAD2/SMAD3. The possible mechanisms by which the *MSTN* mutation inhibited SMAD2/SMAD3, activated *TET1*, and increased myogenesis-specific gene expression, subsequently inducing myogenic differentiation, are shown in Figure 7.

## Materials and Methods

### Ethics statement

All animal procedures were reviewed and approved by the Committee on the Ethics of Animal Experiments at Inner Mongolia University. All methods were performed in accordance with the relevant guidelines and regulations.

### Animals

The *MSTN ^-/+^* cattle used in this study were a cross of female Mongolian yellow cattle and male *MSTN ^-/-^* cattle, as our previous report 6. Five 24-month-old female *MSTN ^-/+^* cattle and five wild type female cattle were selected for muscle sampling. The cattle were fed as ordinary beef cattle in a local livestock farm.

### Muscle tissue collection

Longissimus dorsi muscles were harvested using a muscle-sampler, and immediately rinsed in 75% ethanol for 3 s, then transferred to phosphate-buffered saline (PBS) (Hyclone, Thermo Scientific, Marietta, OH, USA) containing antibiotics (100 U/ml of penicillin and 100 mg/mL of streptomycin) (Gibco-BRL, Grand Island, NY, USA). The samples were then immediately transferred to a biological safety cabinet (HF-1100, HK, China) for cell isolation. Part of the muscle was frozen in liquid N_2_ for RNA-Seq, while the rest of the samples were fixed in 4% paraformaldehyde for tissue slice preparation.

### Histological preparation

For morphological analysis, the Longissimus dorsi muscle tissues from *MSTN* mutant (MT) and wild type (WT) cattle were fixed in 4% paraformaldehyde for 24 h, dehydrated using an alcohol-xylene series, and embedded in paraffin wax. The sample blocks were then cut into 5-μm sections using a microtome and routinely stained with hematoxylin and eosin (H&E). Images were captured using a microscope (DS-Ri1, Nikon).

### Cell isolation and culture

The muscle samples were rinsed in PBS containing antibiotics three times, then transferred into 75% ethanol for 15 s and quickly transferred into PBS. The samples were rinsed another three times, cut into 1-3 mm slices, added 1 mg/mL collagenase IV, and plated at 38.5°C with 5% CO_2_ for 2-3 h. After the tissue had dissolved, the mixture was centrifuged at 1500 rpm/min for 5 min to collect the cells. The cells were cultured in Dulbecco's modified Eagle's medium (DMEM) supplemented with 20% fetal bovine serum (FBS) and 10% horse serum (HS) at 38.5℃ with 5% CO_2_. The cells were passaged after reaching 90% confluence.

### Myogenic cells differentiation and immunostaining

When the cultured cells reached 80-90% confluence, myogenic differentiation was induced by low serum medium comprised of DMEM supplemented with 2% horse serum (Hyclone Ltd) for 5 days. Myogenic cells and myotubes were identified by immunostaining, as described previously 37. Myogenic cells were incubated with primary antibodies against MyoD (1:200; Santa Cruz, USA) and myotubes were incubated with primary antibodies against MHC (1:200; Cell Signaling Technology, USA) at 4℃ overnight, then incubated with fluorescence-tagged secondary antibodies (1:500; Santa Cruz, USA) at 37℃ for 1 h. After incubation and mounting, the samples were stained with 4',6-diamidino-2-phenylindole (DAPI) for 20 min. Samples were observed and photographed using a confocal fluorescence microscope (A1R/A1; Nikon, Japan).

### Fusion index calculation and myotube length measurement

Myotubes induced for 3 d were fixed with methanol for 10 min, then dyed with Giemsa dye (G1015; Solarbio, Beijing, China). Images were captured using a microscope (DS-Ri1; Nikon). The degree of differentiation was determined by calculating the fusion index (i.e. the ratio of nuclei in multinucleated cells to the total number of nuclei) 38 and measuring the length of myotubes (myotube lengths were carefully measured by drawing a line along the long axis of the myotube between two myotube tips and converting the pixels into the unit of length in ImageJ) 39.

### Real-time PCR

The RNA from the myogenic cells and myotubes was isolated using an RNAiso Plus kit (9108; Takara), then reverse-transcribed into cDNA using a PrimeScript RT reagent Kit with gDNA Eraser (Perfect Real Time) (RR047A; Takara). We amplified the cDNA using ABI7500 real-time PCR (Applied Biosystems, America) and SYBR Green (RR820A; Takara). The PCR primers are listed in Table S1. The protocol for PCR amplifications was as follows: 95℃ for 30 s, followed by 40 cycles at 95℃ for 5 s, 60℃ for 34 s, and a final melting curve stage. The cycle threshold (Ct) values of the targeted genes were normalized to the housekeeping gene *GAPDH* using the 2^-ΔΔCt^ method 40.

### Western blotting

The cell samples were rinsed with PBS and lysed in 150 μL of ice-cold Radio Immunoprecipitation Assay (RIPA) buffer. The cell lysates were then centrifuged at 4ºC for 30 min at 8000 × *g*. The supernatant was electrophoresed in a 10% SDS-polyacrylamide gel and transferred onto a polyvinylidene difluoride membrane by electroblotting. The membrane was blocked in 5% non-fat milk in Tris-buffered saline with 0.1% Tween-20 (TBST) blocking solution at room temperature for 1 h. The membrane was subsequently incubated with anti-MSTN (sc-134345; Santa Cruz, USA), anti-PAX3/7 (sc-7749; Santa Cruz, USA), anti-MyoD (Santa Cruz, USA), and anti-TET1 (sc-293186; Santa Cruz, USA) monoclonal antibodies at 1:500 in TBST. The membrane was then incubated for 1 h with a horseradish peroxidase-conjugated goat anti-mouse and anti-rabbit secondary antibody at 1:10000 in TBST, followed by detection using the chemiluminescence labeling detection reagent ECL Plus (Thermo Scientific).

### Prediction of CpG island

The CpG islands and CpG sites in the promoters and gene bodies of *MyoD*, *MyoG*, *PAX3*, and *PAX7* were examined using MethPrimer software online (http://www.urogene.org/cgi-bin/methprimer/methprimer.cgi).

### Methylated DNA immunoprecipitation (MeDIP)

MeDIP analysis was performed according to the manufacturer's instructions (ab117133; Abcam, USA). Total genomic DNA was sonicated (three pulses for 10-12 s at level 2 with 30-40 s intervals between pulses while resting on ice) to yield DNA fragments between 200 bp and 1000 bp in length (Sonics Vibra, USA). One microgram of fragmented DNA was heat denatured to produce single-stranded DNA. Immunoprecipitation was performed for 2 h at room temperature using 1 μL anti-5mC antibody, with 1 μL of normal mouse IgG as the negative control. The mDNA was released from the DNA/antibody complex using Proteinase K at 65℃ for 1 h. Finally, the mDNA was captured, eluted, and used for real-time PCR detection. The PCR primers are listed in Table S2.

### Chromatin immunoprecipitation (ChIP) assay

ChIP assay was performed according to the manufacturer's instructions (26157; Thermo Fisher Scientific) using a previously described protocol 41. Briefly, approximately 1 × 10^7^ cells were fixed with 1% formaldehyde and quenched by glycine. The cells were washed three times with PBS, then harvested in ice-cold PBS containing 1% Halt Cocktail. The DNA was lysed and digested using MNase. The mixture was then sonicated on ice to break the nuclear membrane. The resulting lysate was incubated with anti-SMAD2+SMAD3 (ab202445; Abcam, USA) and protein G beads at 4℃ overnight. Normal rabbit IgG was used as a negative control. The beads were washed four times before eluting the DNA using ChIP elution buffer. The elution was incubated at 65℃ for 1.5 h. The DNA was then revived using a DNA purification kit. The purified DNA was assayed by quantitative PCR. Assays were repeated at least three times. The data represent the average values ± standard deviation (SD) of the representative experiments. The sequences of the primers used are provided in Table S3.

### Luciferase reporter

The *SMAD3* cDNA was amplified from the bovine cDNA and inserted into the pCAG-EGFP vector to obtain an overexpression vector of *SMAD3*. The promoter region of the *TET1* gene was amplified from the bovine genomic DNA and inserted into the pGL3-Basic vector (Promega, Madison, WI, USA). For the luciferase reporter assays, HEK293T cells were seeded in 96-well plates and transfected with the indicated plasmids using Lipofectamine LTX Reagent (Invitrogen Life Technologies, Carlsbad, CA, USA). Cells were collected 48 h after transfection. Luciferase activity was measured using the Dual Luciferase Reporter Assay System (Promega).

### Transfection with *TET1* short hairpin RNA (shRNA) and overexpression vector

*TET1* shRNA and control shRNA were purchased form Santa Cruz (sc-154204-SH and sc-108060). The *TET1* overexpression vector pIRES-hrGFPⅡ-TET1 (#83568) was purchased from Addgene. The knockdown and overexpression vectors were transfected using Lipofectamine LTX Reagent (Invitrogen Life Technologies, Carlsbad, CA, USA) according to the manufacturer's instructions. The transfected cells were then induced with low serum medium (2% HS) after 48 h of transfection.

### Statistical analysis

The data of the *MSTN* mutant and wild type myoblasts were statistically compared using t-tests. Results are expressed as the mean ± standard error of the means (SEM). Differences with p-values of less than 0.05 were considered statistically significant.

## Supplementary Material

Supplementary figures and tables.

## Abbreviations

TGF-β: transforming growth factor β
MT: mutant
WT: wild type
CSA: cross-sectional area
DMEM: Dulbecco's modified Eagle's medium
RIPA: Radio Immunoprecipitation Assay
MeDIP: Methylated DNA immunoprecipitation
ChIP: Chromatin immunoprecipitation
shRNA: short hairpin RNA
