# Supplementary material for: MSTN Mutant Promotes Myogenic Differentiation by Increasing Demethylase TET1 Expression via the SMAD2/SMAD3 Pathway
Source: Int J Biol Sci. 2020 Feb 21;16(8):1324–34. doi: 10.7150/ijbs.40551 (PMC7085230; doi:10.7150/ijbs.40551)
Supplement: Supplementary file 1 — Supplementary figures and tables. [file ijbsv16p1324s1.pdf]

Table S1. Primers used for real-time PCR

Gene Name	Sense (5′ to 3′)	Anti-sense(5′ to 3′)
MSTN	GCTGTAACCTTCCCAGAACCA	CAATCAAGCCCAAAATCTCTCC
TET1	CAAAACCAGGTGGCGCTTG	GCAGGCTCTGTTTTATTTCCAC
TET2	AACCAAGCAAATGCGTTCCC	TGGATCCAGGCTCGACCTTA
TET3	CCTACGGTGGTGCTGAGTTT	ATACTCCTTGGGGCCTGGAT
SMAD3	GGAAAAAGGGCGAGCAGAAC	GGGATGGTGATGCACTTGGT
MyoD	TGACCCGTGTTTCGACTCC	GCAGGGAAGTGCGAGTGTT
MyoG	CGAGTGCCCCTTGAAGACA	CCGACTTCCTCTTACACACCTTACA
PAX3	CAAAGCTTACAGAGGCCCGA	GGTCTCTGACAGCTGGTACG
PAX7	GTGCCCTCAGTGAGTTCGAT	CTGACTCCACATCTGAGCCC
MYF5	GCGGAGACGCCTGAAGAA	CATGATAGATGAGCCTGGAAC
GAPDH	AAATGGTGAAGGTCGGTGTGAAC	CAACAATCTCCACTTTGCCACTG

Table S2. Primers used for MeDIP-qPCR

Gene Name	Sense (5′ to 3′)	Anti-sense(5′ to 3′)
MEMyD	CTGAGGCTGGGCAAAGCCAG	CTGTTGCAAAGTTGCAGAGA
MEyoG	TCGAGTGCCCCTTGAAGACA	GGTCCACAGACACCGACTTC
MEPAX3	GCCTTTTTGGGGAGGGACTC	GGCAGCTTCGCTTGGAAATTAT
MMyoD	GAGGGGACCCCAAAGTCAGC	CGGGCACACTCTCCAAATTTCT
MMyoG	GCATGGGAGCCAGCTAGGGG	CCCCCTCTAAGCTGTTGCTG
MPAX3	TAAGGCCCTGAATACCTCGC	TTGGTAAGTGCCAGCGAACT
MPAX7	TTTTATTGTATCTGGCCTCCAGCG	GAGTCCTGGGGGCGAAACG

Table S3. Primers used for ChIP- qPCR

Gene Name	Sense (5′ to 3′)	Anti-sense(5′ to 3′)
ChTET1	ACAAGCACCAAGTCACCACT	GCTATTGAGATGGAAAAATCACACA

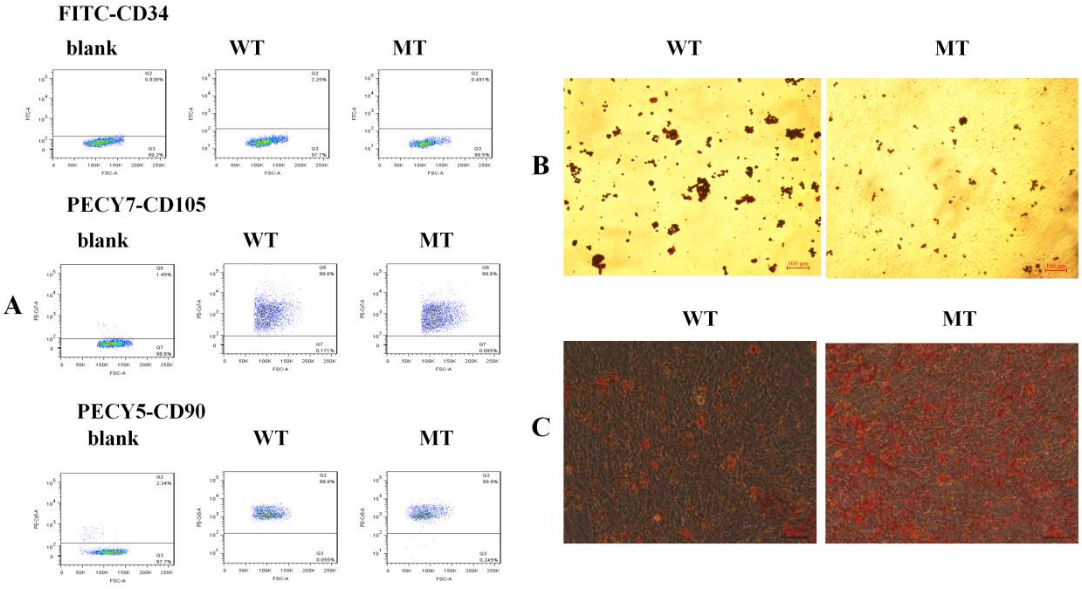

Figure S1. Isolation and identification of the muscle satellite cells from the *MSTN* mutant and wild-type cattle. (A) Percentages of positive cells for CD34, CD90 and CD105, respectively, determined by flow cytometry in muscle derived cells. (B) Adipogenic differentiation and oil red O staining of wild type (A) and *MSTN* mutant (B) satellite cells, results showed a decrease in lipid droplet formation after *MSTN* mutant. (C) Osteogenic differentiation and indocyanin staining of wild type and *MSTN* mutant satellite cells, results showed a increase in calcium formation after *MSTN* mutant. WT: wild type; MT: *MSTN* mutant.

A

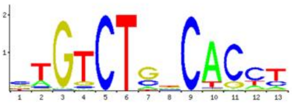

Model ID	Model name	Score	Relative score	Start	End	Strand	predicted site sequence
MA0513.1	SMAD2::SMAD3::SMAD4	10.629	0.858350683410499	458	470	1	GTGCCTGGCACAC

B

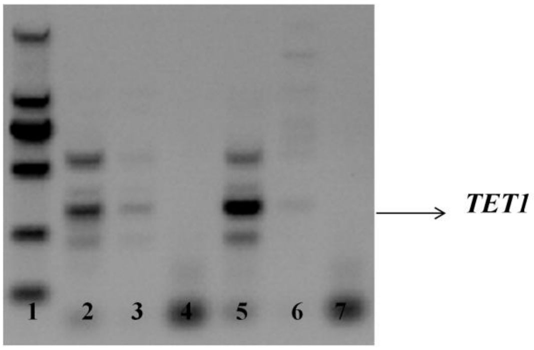

1: DL2000 marker;
2：MT-Input;
3：MT-SMAD2/SMAD3;
4：MT-IgG;
5: WT-Input;
6：WT-SMAD2/SMAD3;
7：WT-IgG.

Figure S2. SMAD2/SMAD3 combined with the promoter of *TET1*. A) The motif of SMAD2/SMAD3/SMAD4 and the predicted results for the combination of SMAD2/SMAD3/SMAD4 with the promoter of *TET1*. B) Amplification of *TET1* by SMAD2/SMAD3 combination. More *TET1* was amplificated with the same amount of ChIP-DNA after *MSTN* mutant.

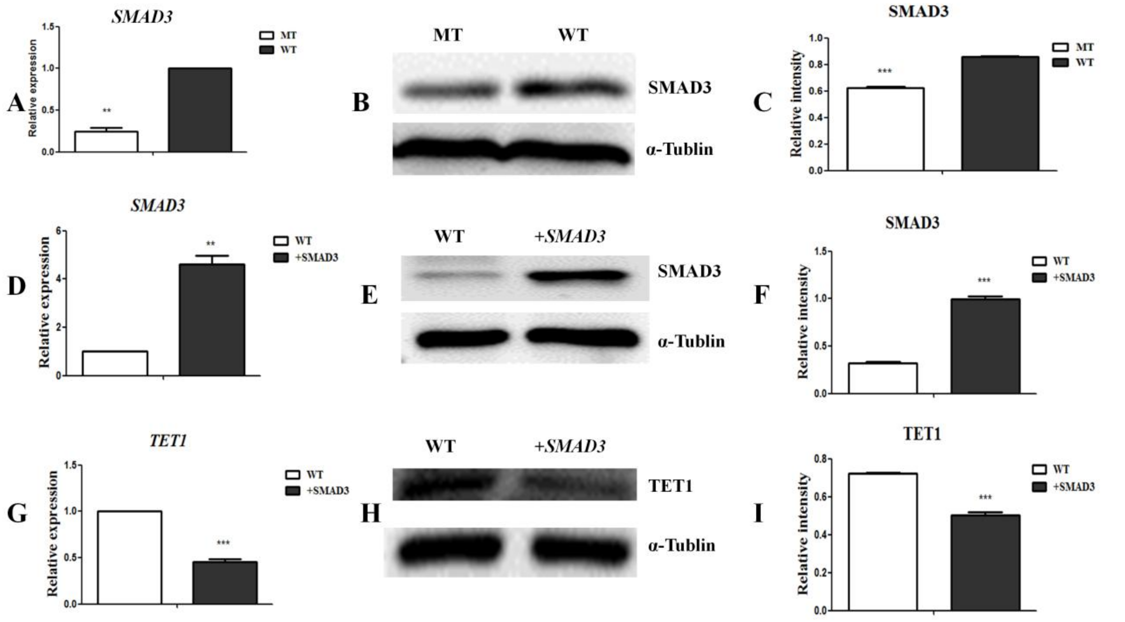

Figure S3. *SMAD3* inhibited *TET1* expression. (A) Expression of *SMAD3* after *MSTN* mutant in mRNA level. (B,C) Expression of SMAD3 after *MSTN* mutant in protein level. (D) Expression of *SMAD3* after overexpression of *SMAD3* in mRNA level. (E,F) Expression of SMAD3 after overexpression of *SMAD3* in protein level. (G) Expression of *TET1* after overexpression of *SMAD3* in mRNA level. (H,I) Expression of TET1 after overexpression of *SMAD3* in protein level.

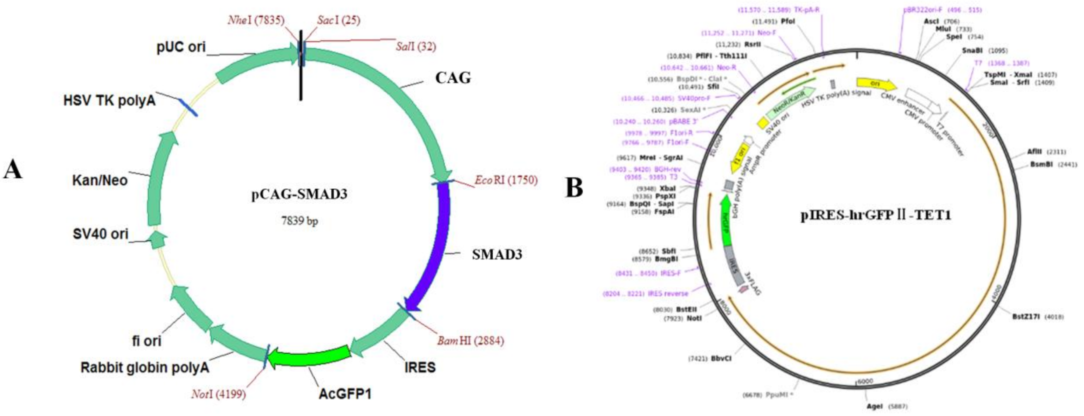

Figure S4. Vectors used for *SMAD3* overexpression (A) and *TET1* overexpression (B).
